# Antiviral Activity and Mechanism of Action of Novel Thiourea Containing Chiral Phosphonate on Tobacco Mosaic Virus

**DOI:** 10.3390/ijms12074522

**Published:** 2011-07-13

**Authors:** Huitao Fan, Baoan Song, Pinaki S. Bhadury, Linhong Jin, Deyu Hu, Song Yang

**Affiliations:** State Key Laboratory Breeding Base of Green Pesticide and Agricultural Bioengineering/Key Laboratory of Green Pesticide and Agricultural Bioengineering, Ministry of Education, Guizhou University, Guiyang 550025, China; E-Mails: fanhuitao0818@163.com (H.F.); bhadury@gzu.edu.cn (B.P.); fcc.jinlh@gzu.edu.cn (L.J.); fcc.dyhu@gzu.edu.cn (D.H.)

**Keywords:** chiral thiourea compounds, tobacco mosaic virus, antiviral activity, mechanism of action

## Abstract

Using half-leaf method *O,O′-*diisopropyl (3-(L-1-(benzylamino)-1-oxo-3- phenylpropan-2-yl)thioureido)(phenyl)methyl phosphonate (2009104) was studied for its activity on tobacco mosaic virus (TMV). It showed good curative activity *in vivo* and the curative activity at 500 μg/mL was found to be 53.3%. *In vivo* treatment with the control agent Ningnanmycin at 500 μg/mL resulted in 51.2% inhibition and curative inhibition rates respectively. Dot-ELISA test was employed to verify the efficacy of activity of compound 200910 for anti-TMV activity. The mechanism of action of compound 2009104 to resist TMV was also studied. The results showed that the resistance enzymes PAL, POD, SOD activity and chlorophyll content after TMV inoculation K_326_ (*Nicotiana tabacum* K_326_) of tobacco plants followed by treatment with compound 2009104 were significantly enhanced. The study of the effect of compound 2009104 on TMV capsid protein (CP) showed that it inhibited the polymerization process of TMV-CP *in vitro*.

## 1. Introduction

Plant virus is a type of plant disease, known as “plants cancer”. In recent years, the impact of climate anomalies and the areas of crops affected by plant virus disease are on the rise resulting in tremendous economic losses in the world in general and China’s agriculture in particular. Plant virus is an obligate parasite in plant cells. Even in the cell nucleus, nucleic acid (DNA or RNA) replication occurs and coat protein is synthesized in the host cell. Since plants do not have the same immune system as animals, and the virus is absolutely dependent on their hosts, to control plant virus disease and develop plant virus inhibitors appears to be extremely difficult. Tobacco mosaic virus (TMV) disease is an important class of common disease occurring in tobacco plants growing all over the world. This virus seriously affects the quality and yield of tobacco. It is estimated that each year an economic loss worth one billion U.S. dollars [1] is encountered worldwide only by the prevalence of TMV disease in agricultural fields. In order to combat TMV, extensive research has been conducted and the most common methods include biological and chemical control. The chemical control method plays an important role in disease prevention because of its simple operation coupled with its economic advantages. As a result, development of efficient, environmentally friendly antiviral agents through chemical synthesis has become the core area of research for eradication of and/or prevention of attack by TMV.

**Figure 1:**
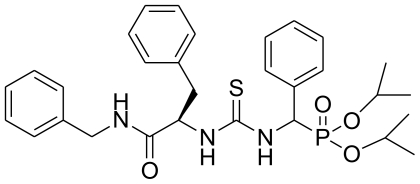
The molecular structure of 2009104.

Thiourea derivatives have a wide range of biological activity on the pesticides which include plant growth regulatory [2], herbicidal [3], antibacterial [4], *etc*. In recent years, certain thiourea inhibitors of plant viruses have also aroused widespread interest in both biological and chemical sectors [5]. In order to create efficient, environmentally friendly antiviral agents, we synthesized a series of chiral thiourea derivatives and evaluated them for anti-TMV activity. The compound *O,O′-*diisopropyl (3-(*L*-1- (benzylamino)-1-oxo-3-phenylpropan-2-yl)thioureido) (phenyl)-methylphosphonate (2009104) (molecular structure shown in Figure 1) displayed high TMV inhibitory activity. In order to investigate the mechanism of action of anti-TMV activity of compound 2009104, herein we studied its effect on defense-related enzymes (PAL, POD, SOD), evaluated chlorophyll content and determined the impact of TMV coat protein. The results after TMV inoculation K_326_ (*Nicotiana tabacum* K_326_) of tobacco plants indicated that the induced resistance was significantly related to enhancement of activity of enzymes PAL, POD, SOD and decrease of chlorophyll content. The influence of TMV-CP showed that the compound 2009104 inhibited its polymerization process *in vitro*.

## 2. Results and Discussion

Development of antiviral agents acting in plants seems to be far more economic when compared to similar research cost for anti-viral agents that act on animals. Nevertheless, mechanistic studies with anti-viral agents for plants require more painstaking research and extensive screening of drugs. The agent being screened can, not only inhibit the proliferation of virus, but also harm the host or even improve other symptoms in the host making the current study a truly challenging one. Mode of action of antiviral agents concerns mainly on the effect of agents on susceptible plants. These include, but are not limited to, whether the agents are susceptible to regulate endogenous hormones; whether the use of biological and chemical factors of treatment plants has any role in viral response of the plants or development of anti-viral factors for local or systemic resistance. To systematically study the mechanism of action with compound 2009104, tests were conducted with tobacco as the main plant. A systematic study on the role of compound 2009104 offering resistance to TMV by using physiology, biochemistry and molecular biology experiment was carried out.

### 2.1. Inhibition of TMV by 2009104

The results of curative, protective and inactivation efficacies of compound 2009104 at a dose of 500 ug/mL against TMV are given in Table 1. High infection inactivation of inhibition rate (84.9%) was noticed *in vivo*; infection curative inhibition rate was 53.3% *in vivo*, the protective inhibition rate was 58.9%. The results showed that compound 2009104 possessed better inhibitory activity against TMV.

The results fully demonstrated that the effect of compound 2009104 for viral replication and proliferation was strongly inhibited, but its mode of action and role of the locus are not clear. The inhibitory effect of the compound on the performance of viral RNA in blocking viral replication or protein synthesis, or the virus shell strip or assembly process, needs further investigation. It is worth mentioning that, although with different pharmaceutical treatments, compound 2009104 can reduce TMV *in vivo* concentrations, there are large differences in the control effect of each treatment. This may be seen from the view point of the mode of action of compound 2009104 which is not single, but multifaceted. It incapicates the virus from invading outside the body and can also repress the virus to breed within the body, while playing a protective role on the host plants. However, the exact pathway through which these roles are performed is still unclear and needs further study.

### 2.2. Dot-ELISA Test to Verify the Efficacy of Compound 2009104 for Anti-TMV Activity

**Figure 2:**
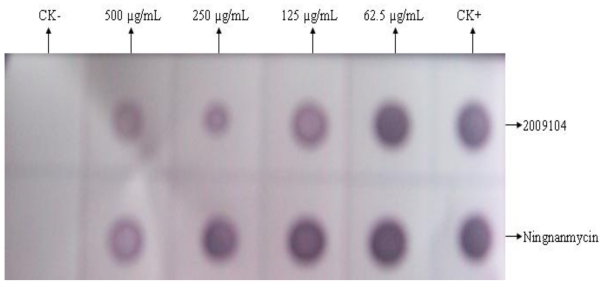
The inhibitory effect of compound 2009104 on TMV in tobacco assayed by Dot-ELISA test.

According to the results shown in Figure 2, TMV content in tobacco leaves treated by compound 2009104 decreased, indicating that compound 2009104 may inhibit replication of TMV in plant along with the compound that inhibits the effect of increasing the concentration. The same observation as with the effect of the previous compound 2009104 that inhibits the activity of TMV is noted.

### 2.3. Effect of Compound 2009104 on Tobacco Defense Enzymes

#### 2.3.1. Effect of 2009104 on PAL Activity in Tobacco Leaves

**Figure 3:**
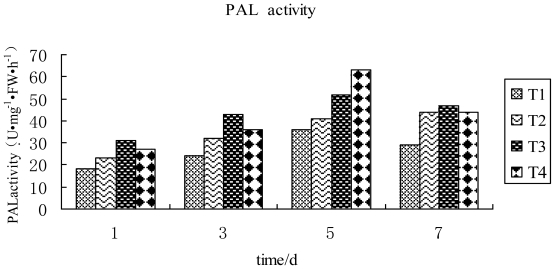
PAL activity in tobacco leaves in differential treatment groups T1: CK; T2: TMV; T3: TMV+ Ningnanmycin; T4: TMV + 2009104.

It can be seen from Figure 3 that after TMV inoculation, followed by spraying with compound 2009104, PAL activity in tobacco leaves during 1~5 d increased and reached a maximum on the 5th day and dropped during 5~7 d. PAL activities were found to be higher than those in which tobacco leaves were inoculated by TMV and treated with water during 1~7 d. From the above results, it may be speculated that compound 2009104 may induce enhancement of PAL activity to promote plant substances in the metabolism of phenylalanine to generate sufficient phenolic compound, lignin and other secondary material. This would improve disease resistance of plants and resist infection of virus particles thereby significantly lowering the symptoms.

#### 2.3.2. Effect of Compound 2009104 on POD Activity in Tobacco Leaves

**Figure 4:**
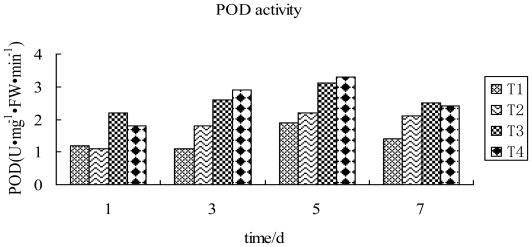
POD activity in tobacco leaves in differential treatment groups T1: CK; T2: TMV; T3: TMV+ Ningnanmycin; T4: TMV+2009104.

It can be seen from Figure 4 that TMV inoculation of tobacco leaves, followed by spraying with compound 2009104, led to an increase in POD activity in tobacco leaves during 1~5 d and reached a peak value on the 5th day. Nevertheless, the activity showed a declining tendency as time progressed from 5th to 7th day. The value during this period was observed to be higher than the one inoculated by TMV and water treated. In TMV inoculated leaves being sprayed with compound 2009104, POD activity was 50% higher than the one being inoculated by only TMV and 73.7% more compared to the one treated with water and 6.5% higher than the one being sprayed with Ningnanmycin on the 5th day. It is obvious from these results that 2009104 is able to interact with the tobacco virus and stimulate the increase of POD activity, causing marked enhancement of plant resistance against various diseases. The virus infection is greatly inhibited by this mechanism.

#### 2.3.3. Effect of 2009104 on SOD Activity in Tobacco Leaves

**Figure 5:**
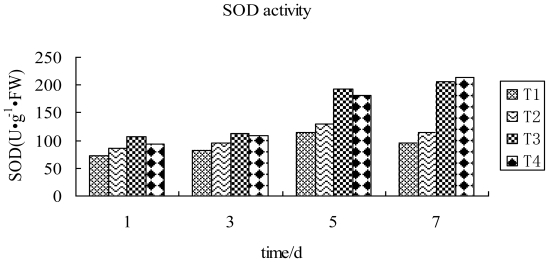
SOD activity in tobacco leaves in differential treatment groups T1: CK; T2: TMV; T3: TMV+ Ningnanmycin; T4: TMV+2009104.

It can again be seen from Figure 5 that TMV inoculation of tobacco leaves, followed by spraying with compound 2009104, changed the SOD activity in the leaves only marginally during 1~3 d, then increased during 3~7 d attaining the highest value on the 7th day which was higher than that inoculated by TMV and CK treatment groups during 5~7 d. In TMV inoculated leaves being sprayed with compound 2009104, SOD activity was found to be higher (87.7%) than that inoculated by TMV. The value was 125.3% higher compared to water treated and 3.4% higher than the one being sprayed with Ningnanmycin on the 7th day after TMV inoculation. From the above results we can conclude that compound 2009104 can increase tobacco SOD activity and enhance the host activity in terms of scavenging of reactive oxygen species, thereby reducing the amount reactive oxygen responsible for cell damage. This eventually leads to an improvement of disease resistance in tobacco plant.

### 2.4. Effect of 2009104 on the Chlorophyll Content in Tobacco

**Figure 6:**
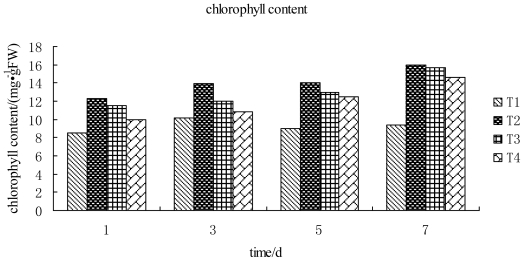
Changes of chlorophyll content in leaves treated with compound 2009104 T1: TMV; T2: CK; T3: TMV+Ningnanmycin; T4: TMV+2009104.

Photosynthesis is a special, and the most basic, life process of green plants providing them necessary growth and energy. Chlorophyll is the photosynthetic organelle of plants whose content is closely related to photosynthesis, extent of viral infection in plants leading to proliferation, expansion and destruction of the plant chloroplasts and factors retarding the synthesis of chlorophyll causing leaf chlorosis and mosaic. Studies have shown that the photosynthetic pigment content is significantly reduced in TMV infected leaves and can lead to severe influenza illness [6]. It can be seen from Figure 6 that after TMV inoculated leaves are sprayed with compound 2009104, chlorophyll content of tobacco increased during 1~7 d and reached the highest value on the 7th day, being higher than the one inoculated with TMV but lower compared to water treated during 1~7 d. More careful inspection of Figure 6 shows that TMV infection of tobacco lowers the chlorophyll content, but the tobacco leaf treated by the compound 2009104 showed an enhancement in chlorophyll content indicating that 2009104 may destroy the virus of the tobacco thereby enhancing the host’s resistance to disease. On the other hand, the chlorophyll content of tobacco leaves treated by compound 2009104 in each treatment period was found to be lower than in the healthy control, indicating that compound 2009104 may not completely suppress the virus damaged by the chloroplast.

### 2.5. Effect of Compound 2009104 on TMV-CP

#### 2.5.1. Identification of TMV-CP

**Figure 7:**
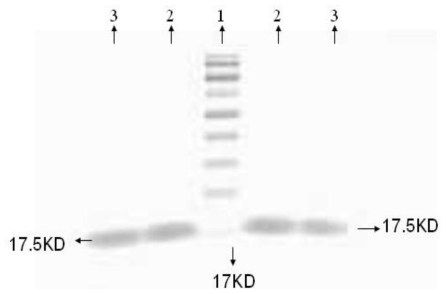
SDS-PAGE of TMV-CP Lane 1: Protein molecular weight marker; Lane 2: TMV-CP; Lane 3: TMV-CP.

The experimental results of TMV-CP extracts identified by SDS-PAGE electrophoresis, are shown in Figure 7; using 12% SDS-PAGE electrophoresis and Coomassie blue staining only molecules with molecular weight 17.5 Kda were seen without any hybrid protein in the lane. It is thus evident that we can obtain pure TMV-CP by using acetic acid extraction method.

Plant virus coat protein plays a very important role throughout the life of the virus. Antiviral agents acting on the virus coat protein can interfere with any one of the steps of virus reproduction in the plant. Previous studies have shown that infection of free nucleic acid of TMV to host, accounts only up to 1% of a complete virus particle in terms of infection activity, while the viral nucleic acid which is not assembled cannot effectively spread over long distances in the host body. Therefore, the virus nucleic acid interaction with the viral coat protein assembled into a complete virus particle is an important part of virus to maintain normal infection activity and long-distance transmission in the host body. As a first step in virus assembly, viral coat protein must be a certain type of polymer that can further fit to the assembly of viral RNA to make a complete virus particle. If the antiviral drugs interfere with this process, there may be an impact on subsequent assembly steps. Consequently, the virus cannot spread in the host body thus inhibiting the virus infection activity. This eventually leads to the desired antiviral effect [7]. Therefore, whether the compound interferes with the polymerization process of the virus capsid protein, has become an important part of study to establish the mechanism of action with the antiviral compound 2009104.

**Figure 8:**
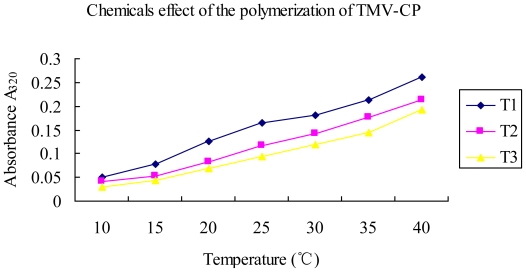
UV (320 nm) absorption of TMV coat-protein polymerization *in vitvo* treated with compound 2009104 at different time intervals T1: TMV-CP; T2: 2009104 +TMV-CP; T3: Ribavirin +TMV-CP.

It can be seen from Figure 8 that compound 2009104 has significant effect in tobacco mosaic virus *in vitro* polymerization process. TMV-CP combines the compound 2009104 in its UV absorption at 320 nm. With the increase in temperature, the trend in the change of value was far less pronounced than the control. According to Smith, KM and other researchers, the observed absorbance of TMV-CP at 320 nm is directly proportional to the degree of polymerization with itself, whereas the degree of change of TMV-CP *in vitro* polymerization is proportional to the temperature. Thus, it may be derived that the compound 2009104 possesses, to a certain extent, an inhibition effect of TMV-CP *in vitro* polymerization, in comparison with TMV-CP treated with ribavirin. The absorbance at 320 nm of TMV-CP treated by compound 2009104 displayed a moderate upward trend which is indicative of the fact that the efficacy of the compound 2009104 to TMV-CP was less than the control agent ribavirin.

## 3. Experimental Section

### 3.1. Materials

In general, tobacco mosaic virus (TMV) was stored in the laboratory after purification of tobacco propagation K_326_ spare.

Heart leaf tobacco (*Nicotiana glutinosa*), dry spot for the TMV host and seeds were purchased from the Institute of Chinese Academy of Agricultural Sciences tobacco. General Tobacco K_326_ (*Nicotiana tabacum* K_326_) for the greenhouse propagation and the seeds were purchased from the Institute of Chinese Academy of Agricultural Sciences tobacco.

*O,O′-*Diisopropyl(3-(*L*-1-(benzylamino)-1-oxo-3-phenylpropan-2-yl)thioureido) (phenyl)methyl phosphonate (2009104) was provided by our laboratory; 2% Ningnanmycin AS was provided by Heilongjiang Qiangr Biochemical Technology Development Co., Ltd.

The 0.45 μm nitrocellulose membrane was purchased from PALL Gelman Laboratory Co., Ltd. While Horseradish peroxidase labeled goat anti-rabbit secondary antibody, NBT and BCIP was procured from Beijing Solarbio Science & Technology Co., Ltd. and TMV antibody was supplied by Agdia Co., Ltd.

### 3.2. Experimental Methods

#### 3.2.1. Anti-TMV Activity of 2009104 [8]

##### 3.2.1.1. TMV Purification

Gooding method was used for the purification of test virus [9], stored at 4 °C for later use.

##### 3.2.1.2. Screening of *in Vivo* Treatment

Five to six leaf spots of blight host of similar growth were selected. First, sap fraction treated leaves inoculated with the virus were painted, applied 500 μg/mL of the test compound and Ningnanmycin in the left lobe after 2 h, right half of the leaf was treated with the solvent as control. Three samples from each treatment group (total number of recorded leaves were 15) were repeated thrice and investigated the number of host dry spots after the spots were completely dry. Finally, lesion inhibition was calculated.

##### 3.2.1.3. Protective Effect of *in Vivo* Screening

Five to six leaf spots of blight host of similar growth were selected; a coating was gently applied with a brush in the left lobe at 500 μg/mL for the test compound and Ningnanmycin. The solvent coating was applied in the right lobe as control. The handing of virus inoculated leaf of sap fraction was done after 24 h, each dealing with three (total 15 leaves were recorded) and being repeated 3 times. The number of host dry spots was investigated after the spots were completely dry, and then lesion inhibition was calculated.

##### 3.2.1.4. Screening *of in Vivo* Inactivation

Five to six leaf spots of blight host of similar growth were selected; a dose of 500 μg/mL for each test compound and Ningnanmycin was mixed with the virus sap at 1:1 (v/v) of passivized sample for 0.5 h. Mechanical inoculation was used to handle left leaves, the virus sap and solvent 1:1 (v/v) fraction in the right half of the leaves were inoculated in each of the three treatment groups (total 15 leaves were recorded) and repeated 3 times. The number of host dry spots was investigated after the spots were completely dry, and then lesion inhibition was calculated.

$$\begin{array}{l} \text{Inhibition }(\%)=[(\text{the number of dry spots for control}-\text{the number of necrotic lesions}\\ \text{treatment})/\text{the number of}\;\text{necrotic lesions control}]\times 100\% \end{array}$$

#### 3.2.2. Dot-ELISA Detection of 2009104 Treated Ordinary Tobacco Inoculated by TMV

By modifying the work of Wu [10] and other similar approaches and taking nitrocellulose positive pencil of 1cm square in each grid point sample (solution was treated by 2009104 tobacco tissue supernatant), each sample (10 μL) was dried on the film using 10 mL 2% skim milk in blocking solution at 4 °C (shaker) for 30 min coupled with TMV anti-body in 10 mL 2% skim milk blocking solution (1:200) at 4 °C (shaker) for 12 h. The film was removed and washed with PBST three times, each time period being of 3~5 min. In addition, horseradish peroxidase labeled goat anti-rabbit HRP secondary antibody in 2% skim milk blocking solution (1:200) was added, the film was immersed at 4 °C (shaker) for 3 h. The film was removed and washed with PBST three times by adding chromogenic substrate solution, each time period being of 3~5 min. After coloring for 10~20 min, healthy leaf tissue supernatant was used as a negative control with brown spots appearing positive, but otherwise negative.

#### 3.2.3. Defense Activity Measurement in TMV Inoculated Tobacco after Treating with 2009104

##### 3.2.3.1. The Extraction of Enzyme

By modifying the work of Xue [11] and other similar approaches, first 1.0 g of different treatment leaves was weighed. Then, 2.0 mL of 0.2 M pH 8.8 sodium borate buffer (containing 5 mM mercaptoethanol, 1 mM EDTA) plus a little Shiying sand was added, ground into homogenate and again sodium borate with 2.0 mL wash buffer was added into the remaining part of the tube. After stirring 20,000 g for 5 min at 4 °C under high-speed refrigerated system, the sample was centrifuged for 20 min. The supernatants as PAL, POD and SOD crude extracts were stored at −40 °C in the freezer.

##### 3.2.3.2. The Phenylalanine Ammonialyase (PAL) Determination

By modifying the work of Zhu [12], the crude enzyme extract was treated with 0.05 M pH 8.8 sodium borate buffer, diluted 5 times with its *L*-phenylalanine as the substrate to 3 mL 0.05 M pH 8.8 by sodium borate buffer and 0.5 mL enzyme solution as the blank control. The instrument was set at zero, UV spectrophotometer was operated at 290 nm and OD_1_ value was measured. The reaction was conducted at 40 °C on a water bath for 5 h, quenched on an ice bath to stop the reaction. At 290 nm, OD_2_ value was measured by subtracting OD_1_ as the net value added for activity. An increase of 0.01 per hour to OD_290_ amount of enzyme activity as a unit of U was obtained, repeated 3 times for each sample.

##### 3.2.3.3. The Superoxide Dismutase (SOD) Determination

The work of Wang [13] was modified by taking four 5 mL tubes (for good transparency), two for the determination of pipe, and the other two for the control tube. The solution was mixed and a control tube was placed in dark, the other tube at 4000 Lux daylight reacted for 20 min (requested by the light pipe in line). At the end of the reaction, the order was not made according to the blank control tube of light; the other tube was measured for its absorbance value.

##### 3.2.3.4. The Peroxidase (POD) Determination

In accordance with the work reported by Wang [13], 5 times of diluted enzyme solution, guaiacol as the substrate and 20 μL of diluted crude enzyme extract were added to a 15 mL test tube containing 3 mL 0.1 M pH 5.8 of 18 mM guaiacol in sodium phosphate buffer. The solution was mixed and balanced uniformly at 30 °C on a water bath for 5 minutes, then added 50 μL of 2.5% (v/v) hydrogen peroxide (H_2_O_2_) solution and mixed evenly to start enzyme reaction with the same volume of distilled water instead of hydrogen peroxide as the blank control, as a zero instrument. After being reacted for 5 minutes, the OD values were measured at 470 nm, per mg protein per minute with the added value of OD_470_ as activity. The experiment was repeated 3 times with each sample.

#### 3.2.4. The Effect of Chlorophyll Content of TMV Inoculated Tobacco after Treatment

According to the work conducted by Peng [14], similarly grown K_326_ was selected. At first, the 7th leaf was identified and a brush was dipped in viral juice. The whole leaf was first inoculated with the virus followed by with a sterile water rinse. Leaves were dried and coated for pharmaceutical facilities. The samples were measured on every second day for their chlorophyll contents. Determination of chlorophyll content: the test samples were cut, weighed and put into 5 mL of 2:1 mixture of acetone and ethanol in the refrigerator at 4 ×C, the dark extraction was carried out for 24 h. The chlorophyll after extraction was placed in a 1 cm thick cuvette, with a 2:1 mixture as reference. The UV spectrophotometer was employed at 645 nm and 663 nm to measure absorbance, chlorophyll content (mg g^−1^ FW) with Ca + Cb.

$$\begin{array}{l} \text{Ca}=12.7\;{\text{A}}_{663}-2.69\;{\text{A}}_{645}\\ \text{Cb}=22.7{\text{A}}_{645}-4.68\;{\text{A}}_{663}\\ \text{Total chlorophyll content}=[20.2{\text{A}}_{645}+8.02{\text{A}}_{663}]\text{V}/1000\times \text{W} \end{array}$$

In the above equation, A_645_ and A_663_ are the absorbance values at the corresponding wavelengths, V is the volume of extraction, W is the leaf fresh weight and chlorophyll content is described in mg·g^−1^.

#### 3.2.5. The Effect of Coat Protein of TMV *in Vitro*

##### 3.2.5.1. Preparation of TMV-CP

Preparation of TMV-CP was executed by dialysis, as described by Fu [15].

##### 3.2.5.2. Effect of Compound 2009104 towards TMV-CP *in Vitro* Polymerization

By following the methods of Jiang [7] and others, initial determination of the TMV-CP, ribavirin and compound 2009104 in soluble potassium phosphate at pH 7.0 buffer was made at temperatures 10 °C and 5 °C as treatment intervals. Each treatment was fully preheated on a water bath and then by using UV spectrophotometer at 320 nm, the corresponding spectra at different temperatures were obtained.

## 4. Conclusions

### 4.1. Efficacy of Compound 2009104

Compound 2009104 is an antiviral agent, has better control effect against TMV in a variety of plant viruses. It can be seen that compound 2009104 plays a direct role in virus particles. It can eradicate the TMV infection thereby protecting tobacco leaves from the control experiments on the dry spots of host of the compound 2009104. In addition to showing good protective and curative effect, curative effect better than antiviral agent Ningnanmycin and protective and inhibition effects close to Ningnanmycin were noted.

Biological method (half-leaf method) and immunological method (Dot-ELISA) were used in this study to determine the virus concentration in the host treated by 2009104. It may be observed from the results that irrespective of the treatment, compound 2009104 could reduce tobacco mosaic virus concentration *in vivo*. The best inoculation was obtained after compound 2009104 was mixed with TMV for 0.5 h. On the other hand, inhibition rate of 84.92%, the lowest control effect with compound 2009104 was found after being inoculated for 2 h.

### 4.2. Effect of Compound 2009104 in Imparting Host Resistance

Most of the preparation and application of plant virus inhibitors generally deal with how resistance to plant disease and its corresponding activity are developed. The present work investigates the role of compound 2009104 towards host plant defense enzymes. The effects were measured by colorimetry and the results are described as follows: Treatment of tobacco leaves infected with TMV by 2009104 showed enhancement of PAL, POD, SOD and other related activities of defensive enzymes to different extents. The increase in PAL, POD and SOD activities in tobacco leaves brought about by the action of the compound enhances resistance against plant disease. The results showed that tobacco leaves treated by compound 2009104 exhibit a variety of physiological and biochemical indicators which are comparable to those of healthy plants. Furthermore, studies were also conducted to understand the effect of compound 2009104 in terms of host chlorophyll content and the outcomes are described below: chlorophyll content was significantly lowered in TMV infected tobacco leaves. Nevertheless, after being treated with compound 2009104, chlorophyll content of the leaves started to rise but remained lower than the control throughout the study. The results reflect that treatment of tobacco leaves by 2009104 can considerably reduce the amount of damage caused by the virus and improve the chlorophyll content, thereby enhancing the host resistance.

### 4.3. Effect of Compound 2009104 on TMV-CP

The study showed that after treatment of the leaves by compound 2009104, the absorbance values of TMV-CP at different temperatures were much lower than the control suggesting that 2009104 may strongly interfere with polymerization process of TMV-CP *in vitro*. The decrease in absorbance may be attributed to the dissociation of capsid protein oligomers by the interaction of compound 2009104 with TMV-CP during the polymerization *in vitro*. Therefore, it may be presumed that compound 2009104 can interfere with the TMV-CP polymerization *in vitro* thereby not only affecting virus assembly but also restricting long-distance transmission of the virus in the host and the extent of viral infection *in vivo*.

### 4.4. Innovation of This Paper

The work demonstrates the first use of Dot-ELISA method to validate high antiviral activity of compound 2009104 by half-leaf method. The results of verification measured by the half-leaf method are fairly consistent. Compound 2009104 has been found to induce resistance against plant disease for the first time. Another noticeable discovery with this compound was that it could interfere with the assembly process of TMV-CP.

## Figures and Tables

**Table 1:** The result of curative, protective and inactivation efficacies of compound 2009104 against TMV.

Pharmacy Name	Average inhibition rate (%)		
	Curative effect	Protective effect	Inactivation effect
2009104 (500 μg/mL)	53.3	58.9	84.9
Ningnanmycin AS (500 μg/mL)	51.2	62.6	88.4
